# How and when fungal endophytes can eliminate the plant growth–defence trade‐off: mechanistic perspectives

**DOI:** 10.1111/nph.18161

**Published:** 2022-05-12

**Authors:** Daniel A. Bastías, Pedro E. Gundel, Richard D. Johnson, Ernesto Gianoli

**Affiliations:** ^1^ AgResearch Limited Grasslands Research Centre Palmerston North 4442 New Zealand; ^2^ Facultad de Agronomía IFEVA Universidad de Buenos Aires, CONICET Buenos Aires C1417DSE Argentina; ^3^ Laboratorio de Biología Vegetal Instituto de Ciencias Biológicas Universidad de Talca Campus Lircay Talca 3480094 Chile; ^4^ Departamento de Biología Universidad de La Serena Casilla 554 La Serena 1700000 Chile

**Keywords:** alkaloids, endophytes, growth–defence trade‐off, phytohormones, symbiosis

## Author contributions

DAB, PEG, RDJ and EG conceived and wrote the study.

## A response to Atala *et al*. (2022) ‘Fungal endophytes improve the performance of host plants but do not eliminate the growth/defence trade‐off’

**Fig. 1:**
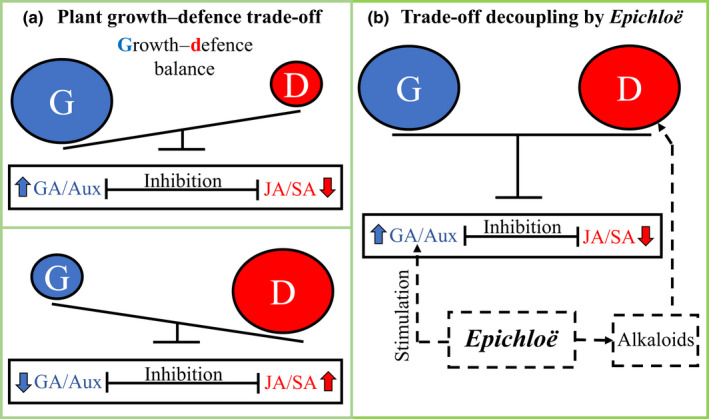
Schematic representation showing the regulation of the plant growth–defence balance by phytohormones (a) and *Epichloë* endophytes (b). (a) The growth–defence trade‐off results from mutual inhibition between growth‐ and defence‐related hormone responses (e.g. gibberellins (GA)/auxins (Aux) and jasmonic acid (JA)/salicylic acid (SA)). (b) *Epichloë* induces plant growth‐related hormones and produces defence compounds (alkaloids) that circumvent the need for defence‐related hormones, thus decoupling the trade‐off. Arrows and truncated connectors indicate positive and negative regulations, respectively. Dashed lines indicate those components and effects associated with *Epichloë* endophytes.

A central paradigm in plant biology is that there is a trade‐off between growth and defence against biotic stresses (Herms & Mattson, 1992; Lind *et al*., 2013; Karasov *et al*., 2017; Züst & Agrawal, 2017; Monson *et al*., 2022). This paradigm is based on recurrent observations that increased production of chemical defences is associated with compromised plant growth, and it provides obvious limits to increasing the productivity of plants that must also resist pests and pathogens (Ballaré & Austin, 2019; Ha *et al*., 2021; Sestari & Campos, 2021). We have recently challenged this paradigm by proposing that fungal endophytes can simultaneously increase plant growth and defence against biotic stresses (Fig. 1) (Bastías *et al*., 2021).

The growth–defence trade‐off largely exists because the hormone signalling pathways that underpin growth and defence are mutually inhibitory. Thus, growth‐related hormones, such as gibberellins/auxins (GA/Aux), repress defence‐related hormones, such as jasmonic acid/salicylic acid (JA/SA), and vice versa (Fig. 1a). *Epichloë* spp. are fungal endophytes of grasses belonging to the subfamily Pooideae that provide an effective defence mechanism to plants through synthesis of alkaloids. Our hypothesis is that *Epichloë* endophytes may uncouple the growth–defence trade‐off by simultaneously inducing plant growth‐related hormones and producing defence compounds (alkaloids) that circumvent the need for host defence‐related hormones (Fig. 1b) (Bastías *et al*., 2021). Our hypothesis predicts that the *Epichloë*‐mediated stimulation of growth‐related hormones will not compromise plant defence despite the downregulation of plant defence‐related hormones following the production of defence alkaloids by endophytes (Fig. 1b).

In a *Letter* published in this issue of *New Phytologist*, Atala *et al*. (2022, pp. 384–387) indicated that we hypothesized ‘a suppression of the growth–defence trade‐off due to the positive effects of endophytes on plant resource status’. As shown in Fig. 1, our hypothesis is based on the plant hormonal control of the trade‐off, not in a resource‐based trade‐off (Bastías *et al*., 2021). Plant hormones can control the trade‐off between growth and defence regardless of resource availability, as has been demonstrated in genetically modified plants (Campos *et al*., 2016; Guo *et al*., 2018; Li *et al*., 2019, 2022; Major *et al*., 2020; Liu *et al*., 2021; Panda *et al*., 2022). Indeed, resource availability can be important for trade‐offs in general and could play a role in the growth–defence trade‐off, as we acknowledge (Bastías *et al*., 2021). However, current understanding at the mechanistic level indicates that plant hormones play a key role in controlling the growth–defence trade‐off (Karasov *et al*., 2017; Ballaré & Austin, 2019; Monson *et al*., 2022).

Atala *et al*. claimed that our study lacks ‘an unequivocal demonstration of a growth–defence trade‐off among nonsymbiotic (E−) plants in the studied species (which is supposed to be eliminated)’. We agree with the authors in that it would have been ideal to provide evidence that the trade‐off is present in the E− plants. This should have been tested using data such as the concentration of defence compounds and relative growth rate measured in the same studies that included E+ plants. Unfortunately, to our knowledge, these data are seldom reported in studies on grasses and *Epichloë* endophytes with E− plants. The alternative of evaluating the growth–defence trade‐off in E− plants using the same dataset utilized to show the decoupling of the trade‐off by *Epichloë* endophytes (fig. 3 in Bastías *et al*., 2021) would not be reliable, since neither growth nor defence data from E− plants can be standardized. In the studies summarized in fig. 3 in Bastías *et al*. (2021), plant biomass in the E− group was measured only once and at different developmental stages across studies. Single biomass measurements do not provide an accurate estimate of plant growth because the initial biomass is not accounted for. Likewise, plant defence in the E− group was determined from different measurements, such as insect body weight or survival. Combining these plant defence estimates would generate high data dispersion due to the different nature of the response variables. This problem of standardization does not apply to the growth–defence relationship shown in fig. 3 in Bastías *et al*. (2021), where we took advantage of the fact that response variables (growth/defence gains) were calculated from two different treatments within each study, and thus data from both plant functions could be standardized by calculating effect sizes. Considering this limitation on data availability to carry out an analysis with E− plants only, in our study we followed the evidence‐based assumption that the growth–defence trade‐off is ubiquitous in plants (Herms & Mattson, 1992; Züst & Agrawal, 2017), including grasses (Lind *et al*., 2013).

Atala *et al*. tested the growth–defence trade‐off in only one plant–endophyte association. Specifically, they worked with the grass *Hordeum murinum* associated with an unidentified endophyte, certainly not *Epichloë*, which has not been found in *H*. *murinum* (Wilson *et al*., 1991; Afkhami, 2012). They claimed that ‘no evidence of the expected trade‐off elimination predicted by Bastías *et al*. (2021) was found in our study system, based on the fact that increased levels of JA hormone and loline and peramine alkaloids (defence‐related compounds) in both plant biotypes (E+ and E−) were associated with reduced plant growth and reproduction. Because the authors base their claim on their own data, it is relevant to address their analyses and conclusions. First, from a mechanistic standpoint, we believe that the use of estimates of plant reproduction to test for the growth–defence trade‐off is not adequate. Plant growth is the appropriate response variable as it is intimately linked to plant defence responses by the mutual inhibition of growth‐ and defence‐related hormones (Fig. 1). The relationship between growth and reproduction, which is largely a matter of plant resource allocation, can vary under different conditions (Bazzaz & Grace, 1997). In fact, Atala *et al*.'s data show that the slopes in the linear models of E+ plants for growth and reproduction vs JA are seemingly different (−14.9 vs −106.8). Second, concerning the plant growth data in Atala *et al*., it is important to recall that our hypothesis posits that the ability of endophytes to increase growth‐related hormones (and, thus, plant growth) constitutes a mechanism to alleviate the trade‐off. However, it is not clear whether the unidentified endophyte associated with *H. murinum* actually exhibits such an ability since there is some overlap between E+ and E− points in the *y*‐axis in the three defence‐related compounds. There is a tendency for higher growth in E+ plants in the three cases, but the statistical significance of these differences should be provided in full linear models. Third, we are puzzled by the authors’ statement that ‘beneficial fungi can induce the expression of key functional genes in their host plants, affecting hormonal (e.g. jasmonic acid) and biochemical pathways (i.e. related to defence alkaloids such as loline and peramine)’. To our knowledge, both loline and peramine alkaloids are produced by fungal endophytes (almost exclusively by *Epichloë* spp.), not by plants (Bush *et al*., 1997; Schardl *et al*., 2013). Yet, since the authors report loline and peramine in both E+ and E− plants, we have to believe that – although highly unlikely – in their study system these alkaloids are plant‐derived compounds. However, because our hypothesis refers to endophyte‐derived defences (fig. 1 in Bastías *et al*., 2021), this would make their results not applicable to the validation or rejection of our hypothesis on the elimination of the growth–defence trade‐off. Fourth, we think that the appropriate manner to test the trade‐off between growth and defence in E+ plants is measuring actual plant functions, as we did in our analysis using published datasets (Bastías *et al*., 2021), instead of using particular compounds or ‘biomarkers’ that do not always translate into the plant function, such as resistance (Kennedy & Barbour, 1992).

As a final note, it is important to remark that we put forward that endophytes *can* eliminate the growth–defence trade‐off, proving our point with *Epichloë* endophytes but acknowledging that not all endophytes would possess the ability to decouple the trade‐off. Our hypothesis clearly posits that this ability is based on both the induction of plant growth‐related hormones and the production of defence compounds by endophytes (Fig. 1). Therefore, a hypothetical case of verification of the trade‐off in another system, and moreover if it does not include endophytes with this ability, would hardly question our main conclusion. We hope that this academic exchange of ideas on methodological and conceptual issues will help advance understanding of the regulation of plant trade‐offs by endophytes.
